# Differential Efficacy of Novel Antiviral Substances in 3D and Monolayer Cell Culture

**DOI:** 10.3390/v12111294

**Published:** 2020-11-12

**Authors:** Robert Koban, Markus Neumann, Philipp P. Nelson, Heinz Ellerbrok

**Affiliations:** 1 Highly Pathogenic Viruses, Centre for Biological Threats and Special Pathogens, Robert Koch Institute, Seestr. 10, 13353 Berlin, Germany; robertkoban@gmx.de (R.K.); neumannm@rki.de (M.N.); nelsonp@rki.de (P.P.N.); 2 Public Health Laboratory Support, Centre for International Health Protection, Nordufer 20, 13353 Berlin, Germany

**Keywords:** orthopoxvirus, antiviral treatment, 3D cell culture, primary human cells, infection model, host-directed therapy

## Abstract

Repurposing of approved drugs that target host functions also important for virus replication promises to overcome the shortage of antiviral therapeutics. Mostly, virus biology including initial screening of antivirals is studied in conventional monolayer cells. The biology of these cells differs considerably from infected tissues. 3D culture models with characteristics of human tissues may reflect more realistically the in vivo events during infection. We screened first, second, and third generation epidermal growth factor receptor (EGFR)-inhibitors with different modes of action and the EGFR-blocking monoclonal antibody cetuximab in a 3D cell culture infection model with primary human keratinocytes and cowpox virus (CPXV) for antiviral activity. Antiviral activity of erlotinib and osimertinib was nearly unaffected by the cultivation method similar to the virus-directed antivirals tecovirimat and cidofovir. In contrast, the host-directed inhibitors afatinib and cetuximab were approx. 100-fold more efficient against CPXV in the 3D infection model, similar to previous results with gefitinib. In summary, inhibition of EGFR-signaling downregulates virus replication comparable to established virus-directed antivirals. However, in contrast to virus-directed inhibitors, in vitro efficacy of host-directed antivirals might be seriously affected by cell cultivation. Results obtained for afatinib and cetuximab suggest that screening of such drugs in standard monolayer culture might underestimate their potential as antivirals.

## 1. Introduction

Billions of dollars are invested in clinical studies each year for the investigation of novel therapeutic substances, among them antiviral therapeutics against a broad range of different viruses [1,2]. Most of these studies are designed based on preliminary research data from in vitro cell culture studies in monolayer cultures where cells grow in a rather artificial way attached to an artificial plastic surface and with reduced cell–cell contact compared to the in vivo situation [3]. Upstream of the clinical studies, in vitro results are verified in animal models which as living organisms on the one hand represent a more complex system than monolayer cultures. On the other hand, animal models are non-human systems in which the cells may react differently to stimuli and may interact with each other in ways different from cells in humans [4]. In the last few years, several promising alternatives to these preclinical stages have been developed among them different three-dimensional (3D) cultivation methods with human cells [5]. Such cultures represent a more in vivo-like model than standard monolayer cultures. They are also superior to animal models in other aspects regarding their human origin. While the complexity of in vivo models can hardly be imitated and replaced by 3D cultures in the near future, they may be able to display a human-specific cellular response to treatment with different substances [6,7]. Therefore, it is more likely that new physiologically relevant targets for antiviral strategies will be identified using 3D cultures instead of standard monolayers. In this context several studies have been published which show a differential efficacy of compounds tested against virus infections in 3D-cultivated cells [8,9,10,11]. We have shown previously that the antiviral efficacy of the anti-tumor drug gefitinib against *Cowpox virus* (CPXV) infections is strongly enhanced in 3D cultures of primary normal human epithelial keratinocytes (NHEK) compared to the respective monolayer cultures. Gefitinib intracellularly targets the human epidermal growth factor receptor (EGFR) and thus inhibits EGFR-dependent signaling via viral homologs of the epidermal growth factor (EGF), which is essential for poxvirus replication [10]. For example, *Vaccinia virus* (VACV), which encodes the *Vaccinia virus* growth factor (VGF), an EGF homologue, hijacks the EGF signaling pathway to spread more efficiently in vivo as well as in vitro [12].

The real potential of gefitinib as an antiviral therapeutic interfering with this pathway became clear only through the use of 3D cell cultures as a first line in vitro identification tool and would have been underestimated and potentially dismissed by screening in conventional monolayer cultures [10]. This finding therefore may be of great relevance because so far there is tecovirimat as the only FDA-approved treatment option for poxvirus infections [13]. Different orthopoxviruses are genetically highly similar. They are of interest for public health because *Cowpox virus* (CPXV) and *Monkeypox virus* (MPXV) are zoonotic viruses, while there is also a potential risk of *Variola virus* (VARV) being used as a biological weapon [14,15,16]. As another benefit, treatment with gefitinib represents a host-directed antiviral approach which minimizes the probability of viral escape mutations in contrast to the virus-directed tecovirimat where escape mutations have already been shown in cell culture [17,18]. Because gefitinib is already FDA-approved for treatment of specific forms of non-small cell lung cancer (NSCLC), repurposing of this drug would cause significantly fewer costs for clinical trials than approving new compounds [19,20]. Besides gefitinib, which is a first-generation receptor tyrosine kinase inhibitor (RTKI), there are several other FDA-approved EGFR-targeting drugs for treatment of different types of cancer whose antiviral potential still has to be elucidated. Among them, there are RTKIs of the first (erlotinib), second (afatinib), and third (osimertinib) generation which have different binding affinities and specificities for the EGFR. While members of the first generation bind reversibly to the intracellular receptor tyrosine kinase (RTK) domain of wild-type EGFR and receptor forms with activating mutations, substances from the second generation bind the EGFR irreversibly without preference for the mutation state [21,22]. The third-generation members, however, bind preferentially mutated RTK domains in an irreversible manner [23]. Another possibility to inhibit EGFR signaling is represented by approved therapeutic antibodies like cetuximab which bind to the EGFR extracellularly and thus could already prevent the binding of viral EGF homologs and subsequent downstream signaling [24].

In this study, we used different EGFR-targeting drugs which were already FDA-approved for treatment of different types of cancer as potential novel host-directed antiviral substances against poxvirus infections. Studies were performed in 3D cell cultures of NHEK which were, compared to our previous studies, optimized regarding culture format and time to qualify them for high-throughput approaches. To evaluate a possible influence of the culture method on the drug efficacy, as already shown for gefitinib, data from 3D culture were compared to the respective conventional monolayer culture. To analyze whether this effect of cell culture on antiviral activity is a phenomenon specific to just one inhibitor blocking signaling of EFGR or if it is a more general feature, the effect of culture conditions on the cell-targeted antiviral activity was studied with several EGFR inhibitors with different modes of action and compared to virus-directed treatment with tecovirimat and cidofovir [17,25].

## 2. Materials and Methods

### 2.1. Cells and Culture Conditions

Pooled primary normal human epidermal keratinocytes (NHEK; PromoCell, Heidelberg, Germany) from juvenile foreskin were cultivated in keratinocyte growth medium 2 (KGM2 ready-to-use; PromoCell). Cells were cultured at 37 °C in a 5% CO_2_ humidified atmosphere and routinely screened for the absence of mycoplasma contamination by qPCR [26].

### 2.2. Generation of 3D Cell Cultures on Decellularized Biological Extracellular Matrix

Decellularized equine pericardium was obtained from Auto Tissue Berlin GmbH (Berlin, Germany; Matrix Patch) and used as a biological extracellular matrix (ECM) for cell culture experiments. Decellularization of the ECM was achieved by a multistaged treatment with deoxycholic acid [27]. Matrix patches were stored in phosphate buffered saline (PBS) with amphotericin B, streptomycin, and penicillin at 4° C under sterile conditions for 3 to 12 months prior to use in 3D cell culture experiments. Matrix patches have been used in cardiac and vascular surgery since several years and they are free from glutaraldehyde and endotoxin.

For 3D cell cultivation, patches were washed twice with PBS and spread out in a petri dish under sterile conditions. Discs of 5 mm in diameter were punched out with sterile biopsy punches (Imtegra, Rostock, Germany), transferred into 96-well ultra-low attachment plates (Corning, Corning, NY, USA) with sterile forceps and equilibrated at 37 °C in a 5% CO_2_ humidified atmosphere in 200 µL of KGM2 for 6–24 h. Monolayer-cultured NHEK, grown to <90% confluency, were washed with HEPES-buffered saline solution (PromoCell) and trypsinized with the DetachKit (PromoCell) according to standard protocol. After equilibration KGM2 was removed and replaced by 150 µL of fresh KGM2. Then 1 × 10^5^ NHEK cells in 100 µL of KGM2 were added slowly to the matrices to avoid turbulences in the well. After 24 h of cultivation, half of the KGM2 was replaced by 125 µL of fresh medium. 3D cultures were incubated for further 24 h at 37 °C in a 5% CO_2_ humidified atmosphere and were afterwards used for infection studies.

### 2.3. Viruses and Conditions of Infection

The recombinant virus strain CPXV BAC pBRf consists of the full-length CPXV BR (ATCC, #VR-302) genome with a mini-F sequence and a GFP gene integrated into the thymidine kinase locus of the viral DNA [28]. Preparation of virus stocks was performed as previously described [10]. Virus stock was screened for absence of mycoplasma contamination by qPCR.

For infection of monolayer cell cultures, 8 × 10^3^ NHEK were seeded in 250 µL of KGM2 in 96-well white polystyrene microplates with clear bottom (Corning, Corning, NY, USA). After 48 h KGM2 was removed from the wells and cells were infected at an MOI of 0.1 in 125 µL of medium (1.6 × 10^3^ plaque forming units) immediately before antiviral treatment.

For infection of 3D cultures, 48 h post cell seeding, pericardium patches were transferred into a new 96-well ultra-low attachment plate with sterile forceps to separate 3D cultures from residual non-adherent cells. Cells were infected at an MOI of 0.1 in 125 µL of KGM2 (2 × 10^4^ plaque forming units). At this time point 2 × 10^5^ cells were present in the culture as determined by qPCR.

### 2.4. Inhibition Studies

Receptor tyrosine kinase inhibitors gefitinib (#S1025), erlotinib (#S1023), afatinib (#S1011), and osimertinib (#S7297) were obtained from SelleckChem (Houston, TX, USA) and stock solutions of 20 mM (gefitinib), 6 mM (erlotinib), 100 mM (afatinib), and 150 mM (osimertinib) were prepared in sterile filtered DMSO (0.2 µm).

The anti-EGFR monoclonal antibody cetuximab was obtained from SelleckChem (#A2000) as stock solution of 34.3 µM in phosphate buffer saline.

The antiviral substances tecovirimat (#TRC-T137330-5MG) and cidofovir (#MBS578807-2) were obtained from Biozol (Eching, Germany) and stock solutions of 1 mM and 20 mM were prepared in sterile filtered DMSO (0.2 µm) and MilliQ water, respectively.

Working concentrations (2X concentrated) of each substance were diluted in KGM2. For mock controls DMSO, PBS, or MilliQ water were diluted in KGM2.

For inhibition studies five concentrations of each substance were added to the cells in 125 µL KGM2 immediately after infection with six replicates for each concentration. Medium was replaced by 250 µL of fresh KGM2 containing the respective concentration of the antiviral substances 4 h post infection (p.i.) to remove unattached viruses. Cells were harvested 48 h p.i. for analysis by immunofluorescence assay (IFA) (1×) and qPCR (3×) and for determination of cell viability (2×).

The half maximal inhibitory concentration (IC_50_) values for each inhibitor were determined through quantification of viral nucleic acids by qPCR (Section 2.6) and subsequent nonlinear regression analyses with Graph Pad Prism 5.04 software (GraphPad Software, San Diego, CA, USA). For calculation of possible cytotoxic effects cell viability was measured (see Section 2.7.) and half-maximal cytotoxic concentration (CC_50_) values were calculated with Graph Pad Prism 5.04 software by nonlinear regression. Selectivity indices (SI) were calculated by forming the ratio of the respective CC_50_ and IC_50_ values.

### 2.5. Immunofluorescence Assay (IFA)

For IFA analysis, 3D cultures were washed by dipping them with sterile forceps into two PBS-filled 50 mL centrifuge tubes. They were fixed with 500 µL of 4% paraformaldehyde for 1 h at room temperature (RT). After successive dehydration with 15% and 30% sucrose for 2 h each, 3D cultures were embedded in Tissue-Tek (Sakura, Staufen, Germany) and equilibrated for 2 h at RT. After flash-freezing in liquid nitrogen, specimens were stored at −80 °C. Frozen sections of 8 µm were prepared with the cryomicrotome Leica CM1950 and transferred to SuperFrost^®^ Plus Gold adhesion microscope slides (Thermo Fisher Scientific, Waltham, MA, USA).

Immunological staining of the sections, confocal laser scanning microscopy as well as analysis and processing of raw microscopy data was performed as previously described [10]. Proteins were stained with antibodies against integrin beta 1 (ITGB1) (Abcam, Cambridge, UK; #30388, mouse), atypical protein kinase C (aPKC) (Santa Cruz Biotechnology, Dallas, TX, USA; sc-208, rabbit), phosphorylated EGFR (pEGFR) (Abcam; #40815, rabbit), EGFR (Abcam; #52894, rabbit), and KI-67 (Abcam; #16667, rabbit) diluted at 1/100 in PBS with 2% BSA and 0.2% NaN_3_. Antigen–antibody complexes were detected with AF647-conjugated anti-mouse (Cell Signaling Technology, Cambridge, UK; #4410, 1/500) and anti-rabbit (Cell Signaling Technology; #4414, 1/500) antibodies, respectively. Infected cells were visualized by monitoring GFP expression from recombinant CPXV.

### 2.6. DNA/RNA Preparation and Real-Time PCR Assays

Cell harvest, DNA/RNA purification, DNAse digestion, cDNA synthesis, and real-time PCR (qPCR) of monolayer and 3D cultures were performed as previously described [10].

Viral genome equivalents (GE) were quantified by determination of the viral *CPXV086* gene (AF482758, nomenclature CPXV BR) using qPCR and normalized to the human reference gene *MYC* (NM_002467). RNA expression of the late expressed viral target gene *CPXV086* and the human proliferation marker *KI-67* (NM_002417) was determined using specific RT-qPCR assays and was normalized to the transcripts of the human reference gene *MYC*. Primers and probes for real-time PCR assays were previously published [10].

### 2.7. Cell Viability Assay

Cell viability was determined with the ATP-based CellTiter-Glo^®^ luminescent cell viability assay (Promega, Madison, WI, USA). Viability of monolayer cultures was analyzed according to the manufacturer’s protocol but homogenization time on an orbital shaker was extended from 2 to 30 min for efficient cell lysis. For 3D cultured cells, specimens were washed with 200 µL PBS and transferred to standard 1.5 mL reaction tubes containing 110 µL fresh KGM2 with sterile forceps. An amount of 110 µL of the CellTiter-Glo^®^ reagent was added and 3D cultures were homogenized on an orbital shaker at 700 rpm for 30 min at RT. After incubation 220 µL of fresh medium was added to the samples and 200 µL of each sample was transferred into two wells of a 96-well white polystyrene microplate (Corning, Corning, NY, USA). Luminescence was measured on an Infinite plate reader with i-control software (Tecan, Männedorf, Switzerland).

## 3. Results

### 3.1. Transformation of the NHEK 3D Cell Culture Model into a High-Throughput Method Does Not Affect Cellular Characteristics

In previous studies we have shown that inhibition of the cellular EGFR with the anticancer drug gefitinib leads to efficient reduction in CPXV spread in 2D monolayer cultivated cell lines [29] and that this effect is strongly increased in 3D cell cultures of primary human cells compared to the corresponding monolayer cultures [10]. To evaluate the biological relevance and the specificity of the differential efficacy of gefitinib in relation to the cultivation systems, several other drugs with different virus- and host-directed activities were tested in both cultivation formats.

**Figure 1:**
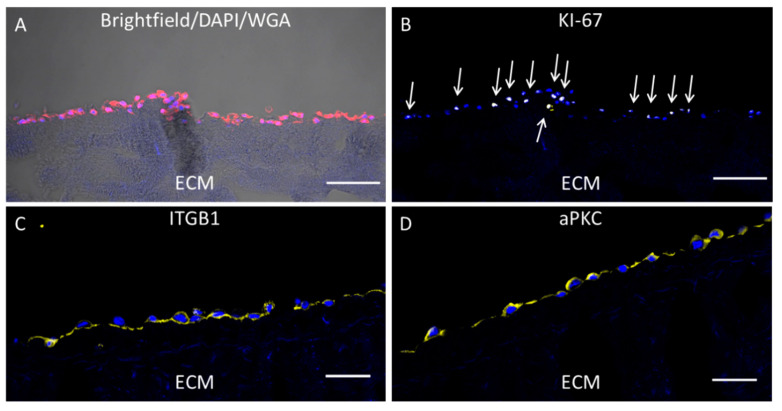
Cultivation of primary human keratinocytes (NHEK) on decellularized extracellular matrix (ECM). The 8 µm sections of ECM were analyzed by microscopy. NHEK grown on decellularized ECM for 2 days were treated with CF594 (red) coupled wheat germ agglutinin (WGA) for staining of sialic acid. N-acetylglucosamin on cell membranes and ECM structure was visualized with a brightfield channel (**A**). After permeabilization, KI-67 (**B**) as a proliferation marker (proliferative cells indicated by arrows), integrin beta 1 (ITGB1) as basal polarization marker (**C**) and the atypical protein kinase C (aPKC) as apical polarization marker (**D**) were visualized with polyclonal antibodies and AF647 (yellow) coupled anti-rabbit secondary antibodies. Cellular nuclei were counterstained with DAPI (blue). Scale bar = 100 µm.

However, the previously published cell culture method using a biological extracellular matrix (ECM) for cell cultivation [10] was time consuming (10 days of cultivation) and not applicable to high throughput screening (24-well plate format). Therefore, as an initial step the dimensions of the 3D cultures were reduced to fit into 96 well plates and the pre-cultivation time of NHEK on the ECM to generate a 3D cell culture before infection with CPXV was reduced from 7 to 2 days. Under these conditions NHEK were in a confluent state for at least 24 h prior to infection. To determine whether growth and differentiation status of the cells were affected by these changes, NHEK were characterized 2 days after seeding onto the decellularized ECM morphologically under the microscope and by immunostaining. NHEK had a square-shaped morphology and displayed a close cell–cell contact (Figure 1A). In vertical cryosections of NHEK the localization of two in vivo epithelial polarization markers was analyzed via immunohistochemistry (IHC) and confocal scanning laser microscopy. While the in vivo epithelial basal differentiation marker integrin beta 1 (ITGB1) was localized towards the ECM (Figure 1C) the in vivo epithelial apical polarization marker atypical protein kinase C (aPKC) was localized towards the apical side of the cell layer (Figure 1D). Sialic acid and N-acetylglucosamine as main components of the cell membranes were stained with AF594 labelled wheat germ agglutinin (WGA) which resulted in a homogenous non-polarized fluorescence signal (Figure 1A). Immunostaining of the cellular proliferation marker KI-67 revealed an overall proliferation rate of about 50% of all cells grown on the ECM (Figure 1B). Besides this elevated proliferation status, the adapted faster and miniaturized culture protocol led to growth characteristics of the NHEK comparable to the previously established cultivation method and was therefore applicable for increased throughput virus inhibition studies.

### 3.2. NHEK 3D Cultivation Does Not Affect the Activity of Virus-Directed Inhibitors

The strongly increased efficacy of gefitinib against CPXV replication observed in 3D culture compared to 2D monolayer culture might be a general effect observable with many antivirals independent of their mechanism of action. To address this question, we analyzed virus inhibition in the two culture systems with the established virus-directed antivirals tecovirimat and cidofovir.

**Figure 2:**
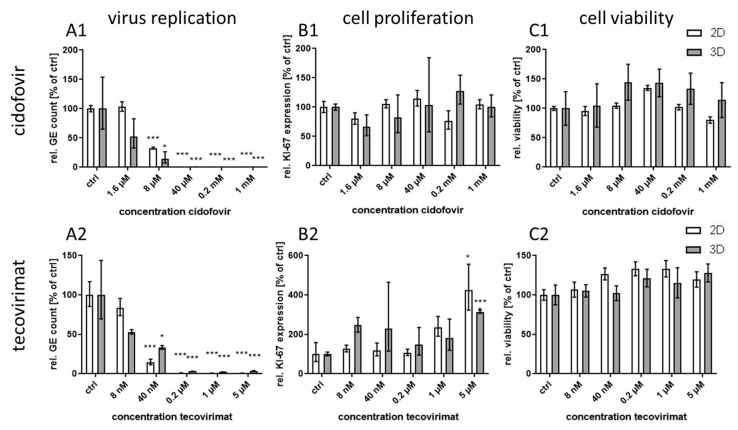
Effect of cidofovir and tecovirimat treatment on *Cowpox virus* (CPXV) infection in primary 3D and 2D cultures. NHEK were grown on decellularized extracellular matrix and infected with CPXV. Infected cells were treated with five concentrations (*n* = 3) of cidofovir or tecovirimat. At 48 h post infection (p.i.) viral genome equivalents (GE) were quantified via qPCR and expressed as GE per cell. All receptor tyrosine kinase inhibitor (RTKI)-treated samples were normalized to infected untreated controls (**A**). Expression of the cellular proliferation marker *KI-67* (**B**) was determined via qPCR, normalized to the cellular reference gene *MYC*, and presented relative to the untreated controls. Statistical significance of reduction relative to the respective controls was calculated by two-tailed Student’s *t*-test (* *p* < 0.05, *** *p* < 0.001). Cell viability was determined by ATP measurement with the cell viability assay CellTiter-Glo^®^ (Promega). Infected but untreated controls (*n* = 2) were taken as 100%. Ratios of treated samples (*n* = 2) to corresponding controls are illustrated as relative viability (**C**).

The two substances were applied separately to CPXV infected NHEK in 3D and corresponding monolayer cultures at five different concentrations covering a range of approximately three logs. At 48 h p.i. NHEK were either used for qPCR analysis or for determination of viability. Both cidofovir and tecovirimat treatments were efficacious against poxvirus infection at comparable concentrations in the two cell culture systems (Figure 2(A1,A2)) suggesting that virus-targeted inhibition is not affected by the cell culture conditions. Cell viability was unaffected by antiviral treatment even at the highest concentrations applied in this study (Figure 2(C1,C2)). In contrast to the host-directed approach with gefitinib, treatment with the virus acting substances did not lead to a reduction in cell proliferation (Figure 2(B1,B2)).

### 3.3. Poxvirus Spread in 3D and 2D Cultures is Differentially Inhibited by Intracellular Blocking of the EGFR via RTKI Treatment

After ruling out that the increased efficacy of gefitinib against CPXV infections in 3D cultures could be a generally observed effect for antiviral approaches, it was examined whether it was a substance specific effect observed for gefitinib or if it was a general phenomenon of the inhibition of the EGFR independent of the specific mechanism of inhibition.

**Figure 3:**
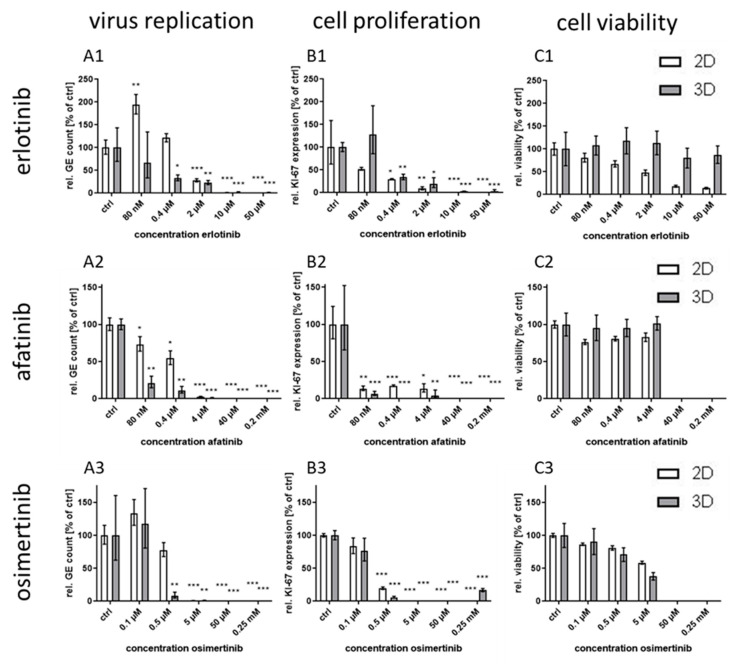
Effect of different RTKIs on CPXV infection in primary 3D and 2D cultures. NHEK were grown either in monolayer or as 3D cultures for 48 h. Cells were infected at an MOI of 0.1 and treated with different RTKI concentrations (*n* = 3). At 48 h p.i. viral GE were quantified via qPCR and described as GE per cell. All RTKI-treated samples were normalized to infected untreated controls (**A**). Expression of the cellular proliferation marker *KI-67* (**B**) was determined via qPCR, normalized to the cellular reference gene *MYC*, and expressed relative to the untreated controls. Statistical significance of reduction relative to the respective controls was calculated by two-tailed Student’s *t*-test (* *p* < 0.05, ** *p* < 0.01, *** *p* < 0.001). Cell viability was determined by ATP measurement with the cell viability assay CellTiter-Glo^®^ (Promega). Infected but untreated controls (*n* = 2) were taken as 100%. Ratios of treated samples (*n* = 2) to corresponding controls are illustrated as relative viability (**C**).

Therefore, further EGFR-targeting drugs with different modes of action and binding affinities to the receptor were applied for virus inhibition studies. In this regard, the EGFR inhibitors erlotinib, afatinib, and osimertinib, each one belonging to another generation of RTKIs, were analyzed for antiviral activity in both culture models. qPCR analyses revealed a significant reduction in viral DNA for all tested substances in both cultivation systems already at the low concentration ranges of the RTKIs (Figure 3A). However, the obtained IC_50_ values diverged in varying degrees between 3D and 2D cultures: while first generation RTKI erlotinib and third generation RTKI osimertinib showed a seven-fold and four-fold IC_50_ reduction, respectively, in 3D cultures compared to the monolayers, the second generation RTKI erlotinib was approximately 20-fold more efficient in a 3D environment (Table 1). The reduction in viral DNA thereby correlated with a decrease in transcripts of the cellular proliferation marker *KI-67*. Decrease in cell proliferation occurred at comparable concentrations in both culture systems (Figure 3B). However, in 2D culture, downregulation of proliferation at 0.4 µM (erlotinib) and, respectively, 0.5 µM (osimertinib) was not sufficient to affect virus replication significantly. Cell viability was differentially affected by erlotinib treatment in 3D and 2D cultures (Figure 3(C1)). While 3D cultures showed unaltered ATP levels even at the highest concentration of 50 µM, the half-maximal cytotoxic concentration (CC_50_) of the RTKI in 2D cultures was already reached at 1.5 µM which resulted in a considerably lower selectivity index (SI) of 1.2 compared to the 3D cultures which showed a SI of >284 (Table 1). In contrast, treatment with afatinib and osimertinib resulted in reduced cell viability in both cultivation systems at comparable concentrations (Figure 3(C2,C3)). While SIs for osimertinib were on a similar and relatively low level of <10, afatinib treatment in monolayer cultures showed an SI of approximately 35 and of >830 in 3D cultures (Table 1).

### 3.4. Efficacy of Extracellularly EGFR-Binding Cetuximab Is Strongly Enhanced in 3D Cultures

To confirm that a virus-induced activation of the EGFR via binding of viral EGF homologs leads to downstream signaling which is required for efficient viral replication, EGFR was not only inhibited at the intracellular ATP binding site but also at the receptor binding site at the extracellular space. In this regard, the EGFR-specific therapeutic antibody cetuximab was applied to CPXV infected NHEK.

**Figure 4:**
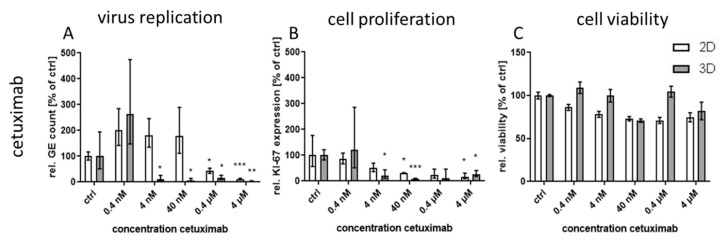
Effect of cetuximab treatment on CPXV infection in primary 3D and 2D cultures. NHEK were grown on decellularized extracellular matrix and infected with CPXV. Infected cells were treated with five concentrations (*n* = 3) of cetuximab. At 48 h p.i. viral GE were quantified via qPCR and described as GE per cell. All RTKI-treated samples were normalized to infected untreated controls (**A**). Expression of the cellular proliferation marker *KI-67* (**B**) was determined via qPCR, normalized to the cellular reference gene *MYC*, and expressed relative to the untreated controls. Statistical significance of reduction relative to the respective controls was calculated by two-tailed Student’s *t*-test (* *p* < 0.05, ** *p* < 0.01, *** *p* < 0.001). Cell viability was determined by ATP measurement with the cell viability assay CellTiter-Glo^®^ (Promega). Infected but untreated controls (*n* = 2) were taken as 100%. Ratios of treated samples (*n* = 2) to corresponding controls are illustrated as relative viability (**C**).

In 3D cultures, virus replication was already significantly reduced at a low concentration of 4 nM whereas in monolayers the first significant drop of viral nucleic acids was observable at a considerably higher concentration of 400 nM (Figure 4A). Thus, CPXV infection was more than 100-fold more sensitive to inhibition with cetuximab in the 3D cell culture model compared to the monolayer culture. According to the treatment with the RTKIs, cell proliferation correlated with the virus inhibition whereby reduction in proliferation in monolayers occurred already at distinctly lower concentrations than reduction in virus genomes (Figure 4B). Cell viability in both culture systems was unaffected by cetuximab treatment (Figure 4C) wherefore SIs could not be calculated.

### 3.5. EGFR Phosphorylation Is Differentially Affected by Intra- and Extracellular Targeting of the EGFR

**Figure 5:**
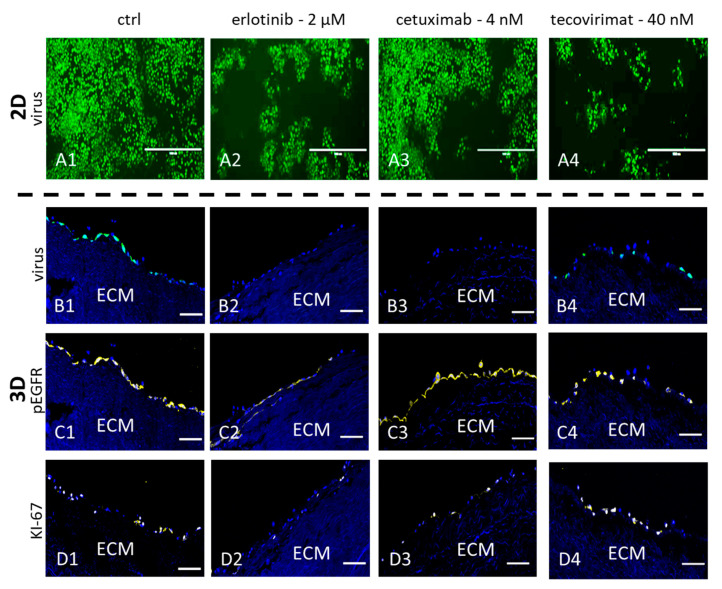
Immunofluorescence assay (IFA) analysis of CPXV infected 2D and 3D cultures treated with different antiviral substances. NHEK cultures were cultured with DMSO as a control (ctrl), 2 µM erlotinib, 4 nM cetuximab or 40 nM tecovirimat and infected with CPXV for 48 h. In 2D cultures and 3D cryosections infected cells were visualized by GFP (green) expression (**A**,**B**). In 3D cultures cellular nuclei were counterstained with DAPI (blue). Phosphorylated EGFR (**C**) and the cellular proliferation marker KI-67 (**D**) were labelled with specific rabbit polyclonal antibodies and visualized with AF647 (yellow) conjugated anti-rabbit secondary antibodies. Scale bar = 100 µm.

To analyze specific activities of the host-directed antivirals, NHEK 3D cultures were infected with CPXV and treated with 1st, 2nd, and 3rd generation RTKIs, and cetuximab, respectively. Vertical cryosections of the cultures were analyzed by IFA for EGFR phosphorylation and KI-67 expression as a cellular proliferation marker, and virus replication. In agreement with the qPCR results, treatment of the 3D cultured cells with the three generations of RTKIs led to a more efficient inhibition of virus spread than in monolayer cultures. Results for the inhibition of virus replication through erlotinib are shown as a representative result for 3D culture in Figure 5(B2) and compared to 2D monolayer culture (Figure 5(A2)). Concentration of erlotinib corresponds to the approximate IC_50_ for the monolayer culture. This was accompanied by a massive reduction in EGFR phosphorylation and cell proliferation in the 3D culture (Figure 5(C2,D2)). Cetuximab also inhibited virus spreading more efficiently in 3D cultures than in 2D monolayer cultures (Figure 5(A3,B3)). While cell proliferation was also downregulated by the treatment (Figure 5(D3)) EGFR phosphorylation remained unaltered (Figure 5(C3)). Inhibition of CPXV replication with the virus-targeting tecovirimat resulted in a comparable reduction in virus spread in both 3D and monolayer cultures (Figure 5(A4,B4)), which was in agreement with results observed by qPCR (Figure 2(A2)). EGFR phosphorylation and KI-67 accumulation in the nuclei was unaffected by tecovirimat treatment (Figure 5(C4,D4)) and thus was on a level similar to the untreated controls (Figure 5(C1,D1)). Comparable results were obtained with cidofovir (data not shown).

## 4. Discussion

In previous studies, we have shown that the antitumor drug gefitinib not only exhibits antiviral activities in a conventional CPXV cell culture infection model [28] but is considerably more efficient against poxvirus infections in a novel three-dimensional cell culture model of primary human keratinocytes than in corresponding conventional monolayer cultures [10]. In this cell culture model, the IC_50_ value for gefitinib was more than 100 times lower than estimated in former monolayer studies and therefore showing antiviral activity in a concentration range which would easily be reached in potential in vivo therapy [29]. In the established 3D cell culture model, cells develop in vivo-like characteristics and therefore it represents an easy-to-handle alternative to far more complex 3D culture methods like epithelial raft-cultures or organ-on-a-chip approaches. In this study, reproducibility, throughput, and time for cell cultivation were further optimized with regard to a standardized screening method for antivirals. These methodical improvements were quite helpful to handle a larger number of 3D cultures in order to decide if the previously observed differential efficacy of gefitinib in 3D culture as inhibitor of CPXV [10] could be assigned also to other potential host-directed antivirals.

In order to make the 3D culture model suitable for high-throughput approaches, the diameter of the pericardium discs used as a biological scaffold was reduced from 8 mm to 5 mm and the pre-cultivation time was reduced from 7 to 2 days. These modifications neither affected cell morphology nor density or polarization. The 3D cultured NHEK in this new format were still infectable with CPXV with similar infection kinetics. Only the rate of proliferative cells in the new culture format was moderately increased compared to the former design, probably due to the shorter pre-cultivation time. However, this had no obvious impact on cells and infection kinetics. With these modifications the costs to set up the 3D culture specimen were reduced by 60% and the time required for testing antiviral substances by 50% (from 10 days to 5 days). Compared to more complex 3D cultures, where pre-cultivation and subsequent infection may take more than 21 days in total [30], the respective savings are considerably higher.

The optimized model was facilitated to examine the specificity of the increased efficacy seen for EGFR inhibition by testing EGFR-targeting RTKIs of different generations. In addition, an inhibitory monoclonal antibody targeting the extracellular domain of the EGFR as another host-directed antiviral was used and compared to the known virus-directed antivirals tecovirimat and cidofovir as controls [25,31]. Compared to the efficacy in conventional monolayer cultures, the treatment with the host-directed substances led to inhomogeneous results. While the IC_50_ value for the second generation RTKI afatinib was—comparable to the former results with gefitinib—significantly lower in 3D than in monolayer cultures, the differences between IC_50_ values for the other first generation RTKI erlotinib was considerably smaller. Even less pronounced was the effect for third generation substance osimertinib where no significant differential response was observed between the culture methods. A possible explanation for this phenomenon might be the different mechanism of binding of the three generations to the EGFR. While the first generation RTKIs reversibly bind to EGFR with strong affinity for both, wild-type and the tumor-associated mutated receptor (L858R), the second generation RTKI afatinib also binds with high affinity to wild-type and mutated EGFR (L858R and T790M), however, this binding is irreversible [21,22]. The third generation RTKI osimertinib also binds irreversibly but with much higher affinity for mutated variants of the EGFR than for the wild-type [23]. This lower affinity for the wild-type receptor should be the main variable in non-cancerogenic normal human keratinocytes between the RTKIs used in this study. This lower affinity therefore might be responsible for an inefficient inhibition of the EGFR in 3D culture and as a consequence of virus replication. However, the reason why inhibition in 2D cultures is not impaired in the same way remains unclear.

Treatment with the monoclonal antibody cetuximab, which binds to the extracellular domain of both wild-type and mutated EGFR variants, led to great differences in virus inhibition between 3D and monolayer cultures similar to first generation RTKI gefitinib. This indicates that EGFR signaling induced through CPXV infection could also be inhibited by targeting the extracellular domain of the receptor. In contrast, treatment of infected cultures with the virus-targeting inhibitors tecovirimat and cidofovir was highly effective in both 3D and monolayer cultured NHEK without significant differences between the culture methods and with IC_50_ concentrations similar to results reported in other studies [8,32]. Taken together, our findings support the assumption that the differential efficacy for virus inhibition of EGFR-targeting drugs revealed in 3D and monolayer cultures is a biologically relevant and pathway-specific phenomenon and not a general feature of more effective virus inhibition in 3D cultivated cells.

IC_50_ values for the EGFR-targeting substances were much lower for 3D than for monolayer cultures (Table 1). While the IC_50_ ratio between 3D and monolayer cultures for afatinib was approx. 20 it was only 7 and 3.7 for erlotinib and osimertinib, respectively. While the difference in efficacy of erlotinib between the cultivation methods was rather small, the selectivity index in 3D culture was increased more than 200 times due to the much higher CC_50_ concentration of this molecule in 3D compared to monolayer cultures. In contrast, for osimertinib CC_50_ was lower in 3D and therefore the SI was also quite low. Nevertheless, except for osimertinib, all tested EGFR-targeting substances, regardless of the mode of action, showed a SI of >250 in infected 3D cultures while SI in monolayer cultures was only between 1.2 and 35. The selectivity indices of the former molecules were comparable to those of the virus-targeting cidofovir and tecovirimat which, however, show similar SIs in both cultivation systems. Therefore, the potential of the host-directed drugs as antiviral substances could only be recognized in the 3D cultures and would be strongly underestimated by conventional cell culture methods.

Since the NHEK in this 3D culture method behave similarly to basal NHEK in human skin regarding morphology, polarization, and gene expression pattern (data not shown) it is plausible that they also behave in vivo-like regarding virus infection and antiviral treatment. Therefore, the data generated in the 3D culture should be more reliable than the data obtained in the conventional monolayer culture. A verification of the observed findings in a mouse model, which normally would be the next step in the pre-clinical course, may not be inevitably closer to the situation in humans because of the species-specific differences between mouse and man. Therefore, a direct transfer of findings generated in primary human 3D cultures to clinical studies in humans might be a realistic aim for the near future. In this respect, a recent study showed that the outcome generated for treatment of metastatic gastrointestinal cancer in patient-derived 3D cell cultures was successfully transferable to the outcome in patients in more than 90% of all cases [7]. Confirmation of the antiviral potential of the investigated EGFR-targeting drugs in clinical studies might open up new opportunities for these substances as broad-spectrum antiviral therapeutic agents. Manipulation of EGFR signaling to facilitate virus entry, replication or evasion of the host immune response also plays an important role for many other viruses, among them HIV, HSV, and HCV [33]. Therefore, blocking of this signaling pathway might also be a promising way to develop host-directed therapies for other viral infections.

In summary, we have verified the important role of EGFR signaling during CPXV infection and shown the advantage of 3D cultures for the identification of potential antiviral components. Further, using the 3D culture system we could show for the first time the potent antiviral activity of the RTKIs erlotinib and afatinib and of the therapeutic monoclonal antibody cetuximab. This potential would have been drastically underestimated by conventional monolayer cultivation. Our optimized 3D culture system now allows efficient and reproducible high-throughput screening of novel potential antiviral substances under more in vivo-like conditions, which could be of particular interest for the study of host directed antiviral approaches.

## Figures and Tables

**Table 1:** IC_50_s, half-maximal cytotoxic concentration (CC_50_)s and selectivity indices (SIs) of tested antiviral substances in CPXV infected primary 3D and 2D cultures ^1^.

Substance	IC_50_ (3D) (nM)	IC_50_(2D) (nM)	Ratio (2D/3D)	CC_50_ (3D) (nM)	CC_50_ (2D) (nM)	Ratio (3D/2D)	SI (3D)	SI (2D)	Ratio (3D/2D)
erlotinib	176	1239	7	>50,000	1500	>33	>284	1.2	>237
afatinib	21	427	20	17,600	15,100	1.2	838	35	24
osimertinib	278	1023	3.7	2700	7500	0.4	10	7.3	1.3
cetuximab	2.5	258	103	>4000	>4000	? ^2^	>1600	>15.5	?
cidofovir	1690	5820	3.4	>1,000,000	>1,000,000	?	>592	>172	?
tecovirimat	11	17	1.5	>5000	>5000	?	>455	>294	?

^1^Bold, underline: more than factor 10 differentially pronounced between 3D and 2D culture. ^2^ ?: not to be calculated.
